# *Macrocystis pyrifera* seaweed extracts combined with *Pseudomonas koreensis* enhance growth and stomatal regulation in the sweet cherry rootstock ‘Colt’ under contrasting water conditions

**DOI:** 10.3389/fpls.2026.1813040

**Published:** 2026-04-22

**Authors:** Macarena Cruzat-Hermosilla, Tamara Alvear, Macarena Gerding, Marcelo Brintrup, Marianne V. Asmüssen, Miguel Garriga, Arturo Calderón-Orellana

**Affiliations:** 1 Departamento de Producción Vegetal, Facultad de Agronomía, Universidad de Concepción, Chillán, Chile; 2 Patagonia Biotecnología S.p.A, Puerto Montt, Chile; 3 Cooperativa de Productividad Forestal, Departamento de Silvicultura, Facultad de Ciencias Forestales, Universidad de Concepción, Concepción, Chile; 4 Centro Nacional de Excelencia para la Industria de la Madera (CENAMAD—ANID BASAL FB210015), Pontificia Universidad Católica de Chile, Santiago, Chile

**Keywords:** Colt rootstock, drought tolerance, macrocystis pyrifera, photosystem II efficiency, plant growth-promoting bacteria, Prunus avium L., Pseudomonas koreensis, seaweed extracts

## Abstract

The Colt rootstock (*Prunus avium × Prunus pseudocerasus*) is widely used in Chilean cherry orchards due to its high compatibility and vigor; however, its low drought tolerance limits its performance in water-scarce environments. Although biostimulants derived from seaweed extracts combined with plant growth-promoting bacteria (PGPB) have shown positive effects in herbaceous crops, their impact on woody species remains largely unexplored. This study evaluated the physiological and morphological responses of young Colt plants to applications of *Macrocystis pyrifera* extracts obtained by alkaline hydrolysis (A1) and mixed hydrolysis (alkaline + enzymatic; A2), applied alone or in combination with *Pseudomonas koreensis* AG 97 (B), including the treatments A1B, A2B, and B alone, under well-watered (WET) and deficit irrigation (DRY) conditions. Under WET conditions, which induced periods of substrate saturation conducive to transient root-zone hypoxia, the A1B treatment increased stomatal conductance by 90% and reduced leaf temperature by approximately 2 °C, mitigating excess-moisture stress and enhancing photochemical efficiency (*Fv*/*Fm*) under low-light conditions, as well as shoot elongation (+23 cm versus 10 cm in the control). Under DRY conditions, A1B reduced stomatal conductance by approximately 70% when plant water potential dropped below −1.1 MPa, limiting dehydration and reducing defoliation to less than 15% compared to 40% in plants treated with B alone, while treatments A1 and A1B also showed higher carbon assimilation rates (>41%). Overall, these results confirm a synergistic effect between *Macrocystis pyrifera* seaweed extracts and PGPB, improving stomatal regulation, physiological resilience, and growth of the Colt rootstock under contrasting water regimes.

## 1 Introduction

Fruit production in Chile has grown steadily over the past three years, with an average annual rate of 3.1%, reaching a planted area of 386,573 hectares. This development has been driven primarily by high-value species, such as cherry (*Prunus avium* L.). The increase in the established area has been concentrated in the south-central regions of the country, reflecting a territorial shift in fruit production (ODEPA, 2025). This change responds to the growing agroclimatic challenges facing the central zone, historically the main productive area, which currently accounts for 77% of the national fruit-growing area (ODEPA, 2025).

In this region, winter rainfall has declined by an estimated 20%-30% over the last decade (MMA, 2024). In addition, climate models project an increase in the number of dry days, intensifying drought conditions. This phenomenon is associated with an increase in the average annual temperature (+4.1 °C), changes in precipitation patterns, and a reduction in freshwater reserves (-39%) (Bambach et al., 2021), creating a challenging scenario for the sustainability of national fruit growing. These conditions have affected the availability of water for irrigation, encouraging the search for climate change mitigation strategies.

Reduced water availability in fruit orchards can lead to moderate to severe water stress during critical stages of the production cycle (Santibáñez et al., 2013). This condition can cause significant alterations in plant physiological and morphological processes, including reduced gas exchange, limited photosynthesis, and decreased biomass production (Moreno, 2009). In fruit agroecosystems, the impact of water stress depends on both the timing and duration of stress. A severe episode during a critical developmental phase, especially if prolonged, can significantly affect vegetative growth and yield (Morales et al., 2013). In cherry trees, severe water stress during the pre-harvest period can reduce pruning weight by up to 40%, indicating a direct effect on plant vigor and productivity (Blanco et al., 2018). This type of stress markedly reduces shoot and leaf growth, particularly in young cherry trees, due to reduced cell division and elongation in meristematic tissues (Catalán-Paine, 2025). In addition, there is an alteration in leaf sugar metabolism, with decreases in sorbitol (41%) and fructose (35%) concentrations, which affects plant energy metabolism (Blaya-Ros et al., 2020).

The response of plants to water stress is directly related to the physiological and morphological characteristics of the rootstock. Under drought conditions, root growth and nutrient solubility decrease, thereby reducing their absorption and transport within the plant (Gunes et al., 2006). This is why rootstocks play a crucial role in water deficit tolerance, as their ability to explore the soil profile and utilize available water depends on their structure and root density. Those considered tolerant have deeper and more efficient root systems, which enable them to maintain water and nutrient uptake under drought conditions (Romero et al., 2006).

Among the most widely used rootstocks in Chile is Colt (*Prunus avium* × *Prunus pseudocerasus*), commonly used in sweet cherry cultivation due to its high compatibility with most commercial varieties and its medium-high vigor (∼ 80% compared to the F12/1 rootstock). However, its shallow root system makes it sensitive to drought, limiting its performance under water deficit conditions. Despite this, Colt has a remarkable tolerance to poor drainage and root asphyxia, making it a suitable option for heavy soils with good water availability (Pino and Hernández, 2014; Catalán-Paine, 2025).

Several studies have shown that, under water deficit conditions, cherry trees grafted onto Colt exhibit a significant reduction in vegetative and productive growth, characterized by a smaller leaf area, shorter shoot elongation, smaller fruit size, and yield that is proportional to the severity of the stress (Blaya-Ros et al., 2020). In contrast, rootstocks such as Mahaleb and Afgano, considered more drought-tolerant (Pino and Hernández, 2014), maintain higher rates of photosynthesis and stomatal conductance under water shortage conditions, which help preserve their physiological activity and yield.

Despite these limitations, the Colt rootstock has been widely adopted in modern fruit growing in Chile. Since 2004, there has been a sustained increase in sales of plants grafted onto Colt, consolidating its position as the most widely planted rootstock in the country in recent years. Between 2008 and 2013, it consistently accounted for more than half of the cherry trees sold annually in nurseries, ranging from 50% to 61% (AGV, 2014). Its use has been concentrated, especially in areas with good water-retaining soils, due to its high compatibility with productive varieties and its resistance to poor drainage and root asphyxia (Ayala, 2009).

Given the scenarios of climate instability and limited water availability, water-use efficiency has become a priority in agriculture. Biomass production is linked to the amount of water available in the soil, and it is estimated that between 50% and 80% of accessible water is used for agricultural activities (Medrano et al., 2007). In this context, the use of biostimulants and beneficial microorganisms is a promising strategy for enhancing crop resilience.

Seaweed-derived biostimulants have been widely reported to enhance plant tolerance to water deficit by improving water-use efficiency, photosynthetic performance, and antioxidant defenses. Several studies using extracts from brown algae such as Ascophyllum, Sargassum, and mixed macroalgal sources have demonstrated positive effects on drought tolerance across different crops, including soybean, wheat, tomato, blueberry, and sugarcane, mainly through improved plant water status, reduced oxidative stress, and enhanced antioxidant responses (Shukla et al., 2018; Alharbi et al., 2022).

Within this broader framework, *Macrocystis pyrifera*–based biostimulants have received increasing attention due to their high polysaccharide content and functional potential. Reports using *M. pyrifera* extracts, alone or combined with plant growth–promoting microorganisms, have shown improvements in germination, root development, and biomass accumulation under stress conditions in crops such as lettuce and maize (Masciarelli et al., 2020; Iparraguirre et al., 2024). The effectiveness of these extracts strongly depends on the extraction strategy, as alkaline hydrolysis is commonly applied to efficiently solubilize cell wall polysaccharides, while combined alkaline–enzymatic approaches allow a more controlled depolymerization, favoring the preservation of bioactive structures associated with biostimulant functionality (Thevanthan et al., 2005). In addition, plant growth–promoting bacteria, such as *Pseudomonas koreensis*, can further contribute to water stress tolerance by producing phytohormones, osmolytes, and enzymes, and by improving soil moisture retention and nutrient uptake (Sati et al., 2021).

Seaweed-derived biostimulants have been widely reported to enhance plant tolerance to water deficit by improving water-use efficiency, photosynthetic performance, and antioxidant defenses. Several studies using extracts from brown algae such as Ascophyllum, Sargassum, and mixed macroalgal sources have demonstrated positive effects on drought tolerance across different crops, including soybean, wheat, tomato, blueberry, and sugarcane, mainly through improved plant water status, reduced oxidative stress, and enhanced antioxidant responses (Shukla et al., 2018; Alharbi et al., 2022).

Within this broader framework, *Macrocystis pyrifera*–based biostimulants have received increasing attention due to their high polysaccharide content and functional potential. Reports using M. pyrifera extracts, alone or in combination with plant growth–promoting microorganisms (PGPB), have shown improvements in germination, root development, and biomass accumulation under stress conditions in crops such as lettuce and maize (Masciarelli et al., 2020; Iparraguirre et al., 2024). The effectiveness of these extracts strongly depends on the extraction strategy, as alkaline hydrolysis is commonly applied to efficiently solubilize cell wall polysaccharides, while combined alkaline–enzymatic approaches allow a more controlled depolymerization, favoring the preservation of bioactive structures associated with biostimulant functionality (Thevanthan et al., 2005).

Although the synergistic application of seaweed extracts and PGPB has shown positive results in herbaceous crops, its effects on woody perennial fruit species such as cherry rootstocks remain unexplored. Thevanthan et al. (2005) suggest that this combination can activate complex signaling networks that improve plant resilience to water deficit. Recent studies in tropical palms confirm this potential, showing that combined biostimulants enhance gas exchange, osmotic adjustment, and antioxidant activity under drought (Rodrigues et al., 2025). The use of these strategies is particularly relevant in Chile, especially for rootstocks such as Colt, which are widely used in traditional orchards and characterized by their low drought tolerance. In this context, the present study aimed to evaluate the effect of applying extracts of *Macrocystis pyrifera*, obtained by alkaline and mixed (alkaline + enzymatic) hydrolysis, in combination with the PGPB bacterium *Pseudomonas koreensis*, on young Colt rootstock plants. The evaluation was conducted under conditions of abundant irrigation and water deficit, assessing physiological and morphological responses across varying levels of water availability.

## 2 Materials and methods

### 2.1 Description of the study site

The study was conducted during the 2025 season, specifically between February and April, in a 65% Rashel mesh shading system located in the nursery of the University of Concepción, Faculty of Agronomy, in Chillán (36°35′46″S; 72°04′49″W), Ñuble Region, Chile. This area has a temperate Mediterranean climate, characterized by hot, dry summers with maximum temperatures exceeding 30 °C and winters with temperatures between 5 °C and 15 °C. Annual rainfall ranges from 800 to 1000 mm, with most falling from May to September (Agrometeorología INIA, 2025).

Two experiments were conducted, using Colt rootstock (*Prunus avium* × *Prunus pseudocerasus*) with an initial growth of 20 cm. The plants were transplanted in January into 2 L pots, using a sandy loam substrate composed of 65% sand, 17.5% clay, and 17.5% silt. This substrate had an organic matter content of 26.5% and was enriched with organic fertilizers containing nitrogen (N), phosphorus (P), and potassium (K) to promote the establishment and initial development of the rootstocks under controlled-shade conditions.

Irrigation was carried out using a micro-sprinkler system, with two inverted medium-range rotary emitters (Aquamic AQ-210, AUTOMAT) per test. This system was installed at approximately 2 meters above ground level, with a 2-meter spacing between emitters, enabling uniform aerial coverage of more than 85% of the substrate surface. Each sprinkler operated at a minimum pressure of 21 psi and a precipitation rate of 5.7 mm h^-^¹, ensuring efficient water distribution during the evaluation period.

### 2.2 Experimental design

Two independent trials were conducted, each in an area of 8 m² (4 m long × 2 m wide), with irrigation management differentiated between them. The WET trial was conducted under commercial irrigation conditions, maintaining the pots at container capacity, corresponding to a volumetric water content of 0.33 m³ m^-3^, which translates to a stem water potential (Ψ_stem_) of approximately –0.5 MPa. In contrast, the DRY trial also began at container capacity. Still, from the sixth week onward, irrigation was applied only when the pots reached 50% capacity, simulating water-deficit conditions under moderate to severe stress (Ψ_stem_ < –1.1 MPa).

Both trials were set up using a completely randomized block design with four replicates per treatment. Each experimental unit consisted of three Colt rootstock plants, grown in individual pots under controlled conditions. Six experimental treatments were established to evaluate the individual and combined effects of seaweed extracts and PGPB on plant physiological responses under both water conditions.

The Control treatment consisted of plants that received no extracts or bacterial inoculation. Treatment B corresponded to inoculation with strain AG-97 (*Pseudomonas koreensis*), obtained from the strain collection of the Laboratory of Agricultural Microbiology, Faculty of Agronomy, University of Concepción. This strain was previously selected from a group of bacterial isolates due to its positive effects on vegetative growth and water stress tolerance in a Prunus sp. rootstock (López-Fernández, 2024).

Treatments A1 and A2 corresponded to the application of *Macrocystis pyrifera* extracts obtained by mixed hydrolysis (alkaline + enzymatic) and alkaline hydrolysis, respectively. The mixed hydrolysis process is protected under patent application WO2025/248437 (SICIT Group), while the alkaline hydrolysis process is patented by Patagonia Biotecnología S.p.A. These extracts differed in their chemical characteristics, as detailed in **Table 1**. The extract obtained through mixed hydrolysis was prepared using an enzymatic treatment based on polysaccharide-degrading enzymes, including alginate lyase and alginate hydrolases, applied under the operational conditions and concentration ranges specified in the patent.

**Table 1:** Chemical composition of *Macrocystis pyrifera* extracts obtained by mixed hydrolysis (A1: alkaline + enzymatic) and alkaline hydrolysis (A2).

Chemical composition of *macrocystis extracts*		
Parameter	A1 (alkaline + enzymatic)	A2 (alkaline)
pH	7 - 7.5	10.5 - 12.5
Alginate Acid (mg kg^-1^)	15,000	2,500
Betaines (mg kg^-1^)	4.5	2.6
Total Carbon (%w w^-1^)	1.5	0.4
Total Nitrogen (%w w^-1^)	0.1	4.0
Mannitol (mg kg^-1^)	1000	700

Finally, combined treatments A1B and A2B included the application of the respective extracts (mixed and alkaline hydrolysis) and the inoculation with AG-97 (*Pseudomonas koreensis*). Bacterial inoculation was performed after transplant (**Table 2**) by applying 10 mL of bacterial suspension at a concentration of 10^6^ colony-forming units (CFU) mL^-1^.

**Table 2:** Application timeline for bacterial inoculation and seaweed extract applications.

Type of application	Application number	Date
Bacterial inoculation	1	Feb. 7^th^
*Macrocystis pyrifera* extract applications	1	Feb. 18^th^
*Macrocystis pyrifera* extract applications	2	Feb. 26^th^
*Macrocystis pyrifera* extract applications	3	Mar. 5^th^
*Macrocystis pyrifera* extract applications	4	Mar. 12^th^

*Treatments that included bacterial inoculation were Bac. (*Pseudomonas koreensis* AG-97), A1B, and A2B (A1B and A2B: seaweed extracts plus bacterial inoculation). Treatments that included seaweed extract applications were A1, A2, A1B, and A2B (A1 and A2: *Macrocystis pyrifera* extracts via mixed and alkaline hydrolysis). The timing of inoculation and extract applications was identical across treatments.

Seaweed extracts were applied via fertigation at a concentration of 0.2% (1.2 mL of extract diluted in 600 mL of water per plant), allowing uniform distribution of the biostimulant through the irrigation system and ensuring adequate availability in the root zone. This application method was selected to promote efficient uptake by the root system and to simulate agronomically relevant fertigation practices. Applications were performed weekly for four consecutive weeks, starting two weeks after transplanting (**Table 2**).

### 2.3 Compatibility between *Macrocystis pyrifera* extracts and *Pseudomonas koreensis*

Before establishing the trials and determining the treatments, a compatibility analysis between *Macrocystis pyrifera* extracts and the PGPB strain *Pseudomonas koreensis* AG-97 was conducted under laboratory conditions. To achieve this, the AG-97 strain was multiplied in peptone standard nutrient broth for 36 hours at 25 °C in an orbital shaker. Once the medium was colonized, the bacterial suspension was standardized to an optical density of 0.2%. Subsequently, 20 µL aliquots of the colonized culture medium were inoculated onto previously sterilized standard nutrient agar plates, mixed with seaweed extract obtained by mixed extraction (alkaline + enzymatic hydrolysis), and onto another plate with standard nutrient agar mixed with seaweed extract obtained by alkaline extraction. Four replicates (plates) were included for each extract type. The plates were incubated in a growth chamber at 25 °C, and bacterial colony growth was assessed.

### 2.4 Environmental conditions and microclimate

Daily data on global solar radiation (W m^-^²), precipitation (mm), relative humidity (%), and air temperature (°C) were obtained from the “Aeródromo Gral. Bernardo O’Higgins, Chillán” weather station, which belongs to the INIA Agrometeorological Network (Chacón and Contreras, 2019; https://agrometeorologia.cl accessed on April 14, 2025), located 3.7 km from the study area. To estimate the effective radiation available for photosynthesis in the experimental area, a portable ceptometer (LP-80, Decagon Devices) was used to measure the Photosynthetic photon flux density (PPFD, µmol m^-^² s^-^¹).

Ten PPFD measurements were taken inside the shade house, approximately 0.5 m above the soil surface, near the plant canopy, at the center, edge, and between rows of each trial. Likewise, 10 PPFD readings were taken outside the shade house, under direct radiation conditions, to calculate the percentage of PPFD intercepted in each trial, determined using the following equation:


$$PPFD\;Internal\;=\;(1\;-\;\frac{PPFD_{in}}{PPFD_{out}})\cdot \;100$$

Where PPDF Internal corresponds to the percentage of photosynthetically active radiation (PPFD) intercepted by the canopy inside the shade house; PPFD_in_ represents the average value of readings taken under shade conditions, at the height of the plant canopy; and PPFD_out_ corresponds to the average value of readings taken in full sun conditions (outside the shade house).

At the beginning and end of each physiological measurement, air temperature and relative humidity were recorded inside the shade house, above the plant canopy in each trial. These variables were measured using integrated sensors from a portable porometer (LI-COR LI-600, LI-COR Instruments, Nebraska, USA). The data obtained were used to calculate the air vapor pressure deficit (VPD_air_) using the equation proposed by Howell and Dusek (1995).


$$VPD_{air}=0.6108\cdot exp(\frac{17.27\cdot T}{T+237.3})\cdot 1-(\frac{RH}{100})\;$$

Where RH is the relative humidity of the air and T is the air temperature.

### 2.5 Plants and soil water

Stem water potential was measured in both trials (WET and DRY) from the sixth week of evaluation, at midday (12:00 to 15:00), using a pressure chamber (Model 615, PMS Instruments, Corvallis, USA), following the protocol described by McCutchan and Shackel (1992). Measurements were taken simultaneously in both trials to monitor the physiological response of plants to water deficit in the DRY trial and to abundant commercial irrigation in the WET trial. The first measurement was taken before the irrigation regime was adjusted in the DRY trial, and subsequent measurements were taken every two weeks. In each experimental unit, a leaf was selected and covered with an opaque, airtight bag at least 40 minutes before measurement to equilibrate water potential between the leaf and the stem.

The volumetric content of the soil was monitored only within the effective root zone of the plants in the second block of both trials. Measurements began in the third week of the trials (end of February) and continued until their completion (beginning of April). Capacitance sensors (model GS1, Decagon Devices, Pullman, WA, USA) installed in the pots at a depth of approximately 10 cm were used for this purpose. The effective root-zone and sensor installation depths were determined based on the previously estimated root development. The data were recorded at 15-minute intervals and stored in a data logger (model Em5b, Decagon Devices, Pullman, WA, USA).

### 2.6 Plant physiology and growth

Stomatal conductance (mmol m^-^² s^-^¹) was determined using a portable porometer (LI-600, LI-COR Biosciences, Nebraska, USA) on three mature leaves located in the upper third of each plant’s canopy. Leaf temperature (°C) was recorded using the infrared temperature sensor integrated in the same porometer (LI-600) on a single mature leaf from the same stratum.

The efficiency of photosystem II (PSII) was evaluated using the *Fv*/*Fm* parameter with a chlorophyll fluorescence meter (Pocket PEA, Hansatech Instruments, Norfolk, UK). One mature leaf per plant was selected from the upper third of the canopy and adapted to darkness for 30 minutes using opaque clips (Reyes-Díaz et al., 2009). Subsequently, minimum (Fo) and maximum (*Fm*) fluorescence values were recorded, and variable fluorescence (*Fv*) was calculated as Fm – Fo. The photochemical efficiency of PSII was then obtained using the relationship *Fv*/*Fm*.


$$\frac{Fv}{Fm}\;=\;\frac{Fm\;-\;Fo\;}{Fm}$$

All physiological measurements were taken simultaneously at midday on mature, healthy leaves without visible damage and were repeated weekly.

During the WET and DRY trials, five measurements of stem growth and leaf fluctuations were taken in each experimental unit. Stem growth was measured from the base of the crown using a manual tape measure to ensure accurate quantification of elongation. Leaf fluctuations, meanwhile, were recorded by counting and direct observation, accounting for changes in leaf number.

### 2.7 Aboveground and root biomass

To quantify aboveground and root biomass, all plants from both trials were harvested at the end of the physiological measurements, when plants reached a height between 36 and 46 cm. This process was carried out with extreme care to avoid damage to plant structures. In the laboratory, the plants were separated into three components: roots, stems, and leaves, ensuring an accurate division between the root system and the stem at the crown level.

Next, the fresh weight of the above-ground structures (stem and leaves) was recorded using a precision balance (± 0.001 g). The samples were immediately stored in airtight bags and refrigerated to prevent moisture loss before dry-weight analysis.

To determine the dry weight (constant weight), the samples were dried in a forced-air oven (Heraeus model D-6450, Hanau, Germany) at 65 °C. Previously, a representative sample was selected for periodic measurements to establish the time required to reach constant weight. Based on this test, the dry weight stabilized after 3 days of exposure. Consequently, all samples were weighed at the end of this period, and their final dry weight was recorded.

Before weighing, root samples were sieved through a No. 16 laboratory sieve (Standard Testing Sieve, USA) to remove adhering substrate residue that could interfere with accurate dry-weight measurement.

### 2.8 Water use efficiency and water productivity

At the end of the physiological evaluations, a composite sample was collected from the leaves of the experimental unit for each treatment in both trials to estimate intrinsic water-use efficiency, measured as the difference in the carbon isotope ratio (δ^13^C). The sampled leaves were mature and fully developed. Once the leaf samples were collected, they were dried in an oven at 65 °C until the sample weight stabilized. After this, the dried samples were ground and sieved to obtain a fine, homogeneous powder. δ^13^C and total carbon percentage (%C) were determined using an EA-GSL gas preparation module (Sercon, UK) coupled to a mass isotope ratio spectrometer (20–22 IRMS, Sercon, UK). Before each analysis, an ultra-high-grade reference gas (Ultra High-Grade CO2, Linde Group) was injected to correct for CO_2_ drift. A calibrated laboratory standard (Corn Flour SCC2256, Sercon, UK) was analyzed every ten analytical samples. One standard sample was checked every ten analytical samples as an internal control of analytical quality. The stable carbon isotope composition (δ^13^C) of each sample was determined using the following equation (Farquhar et al., 1989):


$$\delta 13C\;(‰)=[\frac{{\left(\frac{13C}{12C}\right)}_{sample}-{\left(\frac{13C}{12C}\right)}_{standard}}{{\left(\frac{13C}{12C}\right)}_{standard}}]\cdot 1000$$

Where 13C/12C_sample_ and 13C/12C_standard_ are the measured 13C/12C ratios for the leaf sample and the PDB standard (Pee Dee Belemnite), respectively.

### 2.9 Chemical composition analysis of seaweed extracts

The chemical composition of the seaweed extracts was determined using standardized analytical methods. The pH was measured in a 10% (w/w) solution prepared in demineralized water using a calibrated pH meter equipped with a glass–calomel electrode system, following OECD guideline 122.

Total nitrogen content was determined by catalytic combustion of the sample with subsequent chemiluminescence detection, according to ASTM D8083. Total carbon content was quantified using catalytic combustion followed by infrared detection, in accordance with UNI EN 13654-2:2001.

Alginic acid content was determined spectrophotometrically after derivatization with meta-hydroxybiphenyl (MHPB), following the Chinese standard NYT 3174-2017. Mannitol content was quantified using hydrophilic interaction liquid chromatography (HILIC) coupled with a triple quadrupole mass spectrometer, based on the Helix application note “HPLC Analysis of Sugar Alcohols and Sugars”.

Betaines were quantified by liquid chromatography–tandem mass spectrometry (LC–MS/MS) after solid-phase extraction (SPE), according to the method described by MacKinnon et al. (2010).

All compositional analyses were performed on three independent hydrolysis replicates per extract, and each analytical determination was carried out at least in duplicate. Results are reported as mean values.

### 2.10 Quantification of cultivable *Pseudomonas* spp.

Root samples were collected at the end of each trial, with all utensils cleaned with 70% ethanol. The samples were then stored at 5 °C until transfer to the laboratory, where the total colony-forming units (CFU) of *Pseudomonas* spp. were quantified using a modified method of McSpadden Gardener and Weller (2001).

For microbiological analysis, 1 g of root surface tissue was collected, macerated in a sterile mortar, and transferred to a test tube containing sterile saline (0.89% NaCl). This suspension was kept under constant agitation (150 rpm) at 25 ± 1 °C for 24 hours.

Subsequently, six serial dilutions were prepared in 1.5 mL plastic tubes, each containing 900 µL of sterile saline, and three 10 µL microdroplets of each dilution were seeded onto Petri dishes containing King B (KB) medium. The plates were incubated at 24 °C for 24 hours, and the CFU mL^-^¹ count was performed, considering only those colonies that showed fluorescence under ultraviolet light (TFX-20-M transilluminator; Vilber Lourma).

### 2.11 Statistical analysis

In both trials, the data were subjected to analysis of variance (ANOVA), after verifying the normality of the distribution (Shapiro-Wilks test), homogeneity of error variances (Levene test), and additivity (Tukey test). Differences between means were determined using the LSD test (alpha = 0.05). The relationships among physiological variables, morphological changes, and moisture content were analyzed using linear and quadratic regression. All statistical analyses were performed using RStudio software version 2025.05.0 (RStudio, Posit, USA).

## 3 Results

### 3.1 Environmental conditions and characterization of irrigation

**Figure 1:**
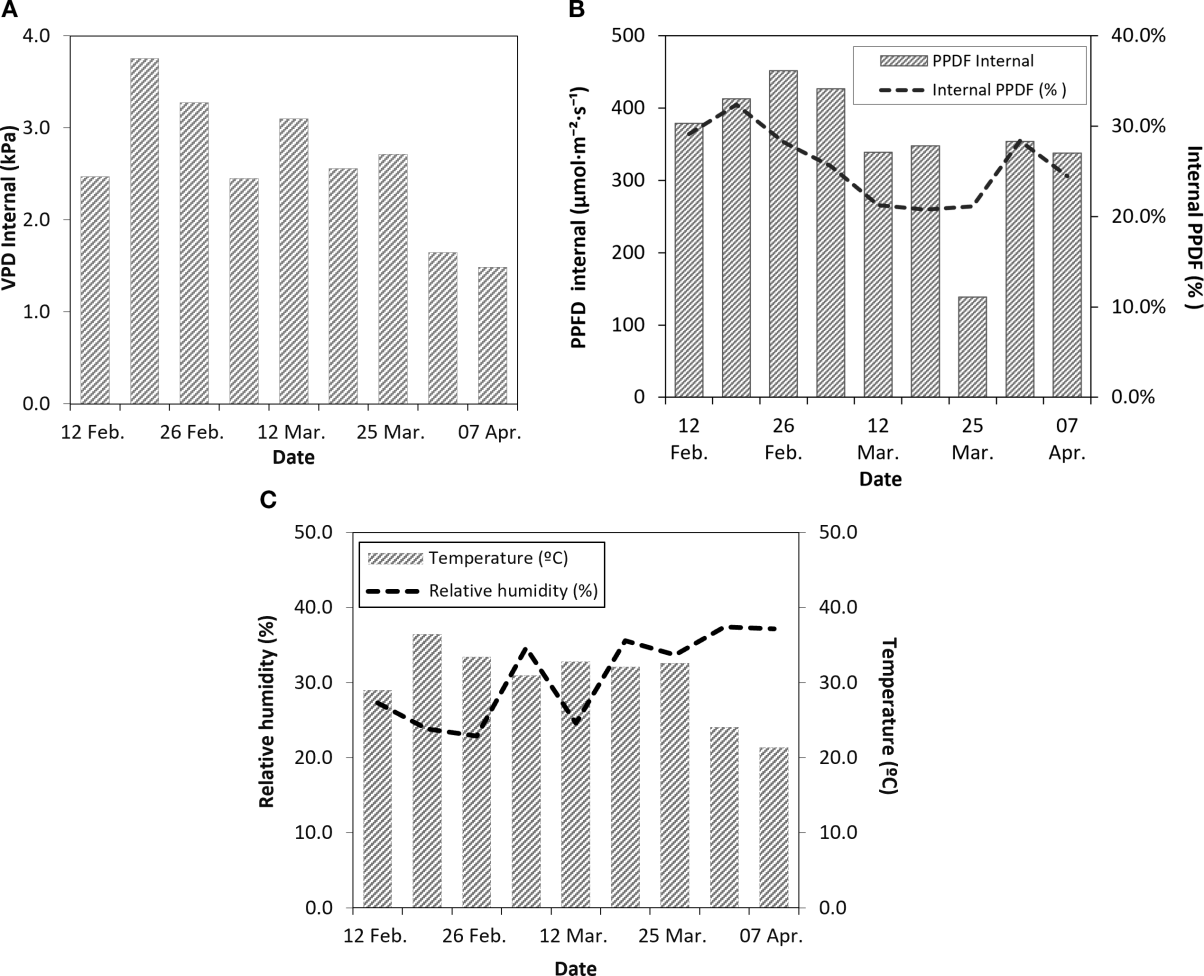
Internal environmental conditions of a shade house located in Chillán, Ñuble Region. **(A)** Internal vapor pressure deficit (VPD), **(B)** Internal photosynthetic photon flux density (PPFD) and its percentage relative to external conditions, and **(C)** Internal air temperature and relative humidity.

During the experimental period (February–April), the microclimatic conditions inside the shade house showed two contrasting phases (**Figure 1**). Vapor pressure deficit (VPD; **Figure 1A**) indicated an initial period of high atmospheric demand during early to mid-February, with values exceeding 3.0 kPa, followed by a progressive decline toward the end of the experiment, when VPD decreased to values below 1.5 kPa. The PPFD inside the shade house (**Figure 1B**) reached its highest values during the first phase of the experiment, exceeding 400 µmol m^-^² s^-^¹, and remained relatively stable over time. An abrupt decrease in internal PPFD was recorded on March 25, associated with a nearby fire event. Throughout the experiment, internal PPFD represented approximately 20–30% of external radiation, with minor temporal fluctuations. Internal air temperature and relative humidity (**Figure 1C**) reflected these microclimatic changes. The early phase of the experiment was characterized by higher temperatures (28–35 °C) and lower relative humidity, whereas the later phase, from late March to early April, showed lower temperatures and increased relative humidity, stabilizing around 35–40%.

**Figure 2:**
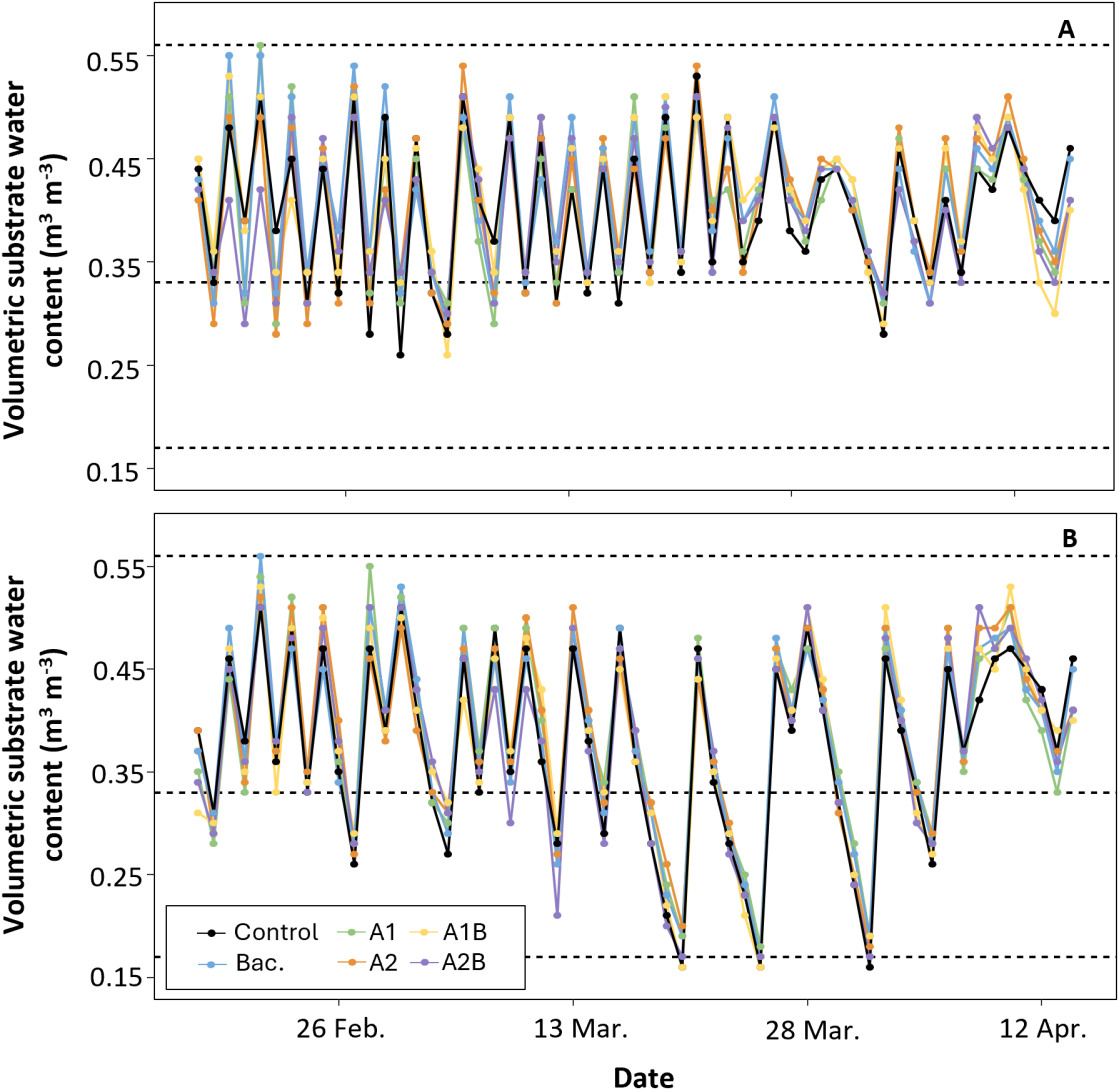
Volumetric water content in the substrate over time in ‘Colt’ cherry rootstocks under two irrigation regimes: **(A)** WET (maintained at container capacity) and **(B)** DRY (irrigated at 50% capacity from the fourth week). The dotted line at 0.33 m³·m^-^³ indicates container capacity, while the lines at 0.17 m³·m^-^³ and 0.56 m³·m^-^³ indicate the permanent wilting point and total porosity, respectively, according to the methodology of Peverill et al. (1999).

Under WET conditions, substrate volumetric water content remained close to container capacity throughout the evaluation period (**Figure 2A**). Moisture values fluctuated between 0.33 m³ m^-^³ and the total porosity of the substrate (0.56 m³ m^-^³), with no sustained periods below container capacity. Temporal oscillations reflected irrigation events, and no consistent differences among treatments were observed, indicating homogeneous water dynamics under abundant irrigation. In contrast, under DRY conditions, soil water dynamics showed three distinct phases (**Figure 2B**). During the first four weeks, volumetric water content followed a pattern similar to that of the WET trial, remaining between container capacity and total porosity. After the onset of deficit irrigation, substrate moisture declined and fluctuated between container capacity and values close to the permanent wilting point (0.17 m³ m^-^³). During this period, irrigation was applied when moisture approached approximately 50% of container capacity, resulting in several events in which the permanent wilting point was reached. Toward the end of the experiment, volumetric water content partially recovered, approaching levels comparable to those observed under WET conditions. Within each irrigation regime, soil water dynamics were similar among treatments.

### 3.2 Physiological responses

#### 3.2.1 Stem water potential

**Figure 3:**
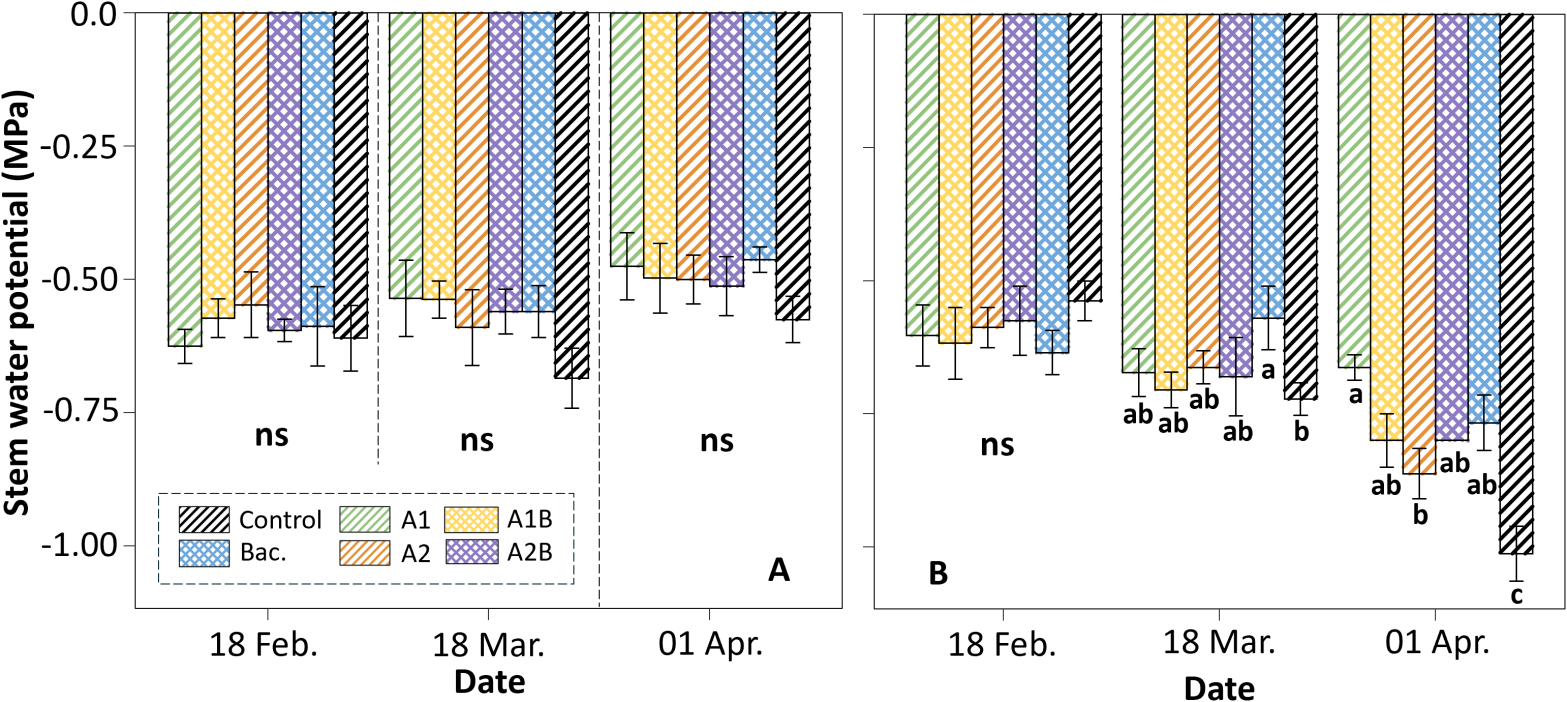
Stem water potential (MPa) measured at midday (12:00–15:00) in cherry rootstock ‘Colt’ under two irrigation regimes: **(A)** WET (maintained at container capacity) and **(B)** DRY (irrigated at 50% container capacity from week four). Treatments included Control (no application), Bac. (*Pseudomonas koreensis* AG-97), A1 and A2 (A1: mixed extraction enzymatic hydrolysis + alkaline; A2: alkaline extraction), and combined treatments A1B and A2B (extracts plus bacterial inoculation). Different letters indicate statistically significant differences between treatments according to Fisher’s LSD test (p < 0.05).

In the WET trial (**Figure 3A**), all treatments showed similar stem water potential (Ψ_stem_) values at the beginning of the experiment, coinciding with the first application of seaweed extracts on February 18. Throughout the evaluation period, Ψ_stem_ remained within a narrow range, and no statistically significant differences among treatments were detected. At the dates when Ψstem reached its lowest values (March 18 and April 1), the control consistently exhibited more negative values compared to the treatments receiving *Macrocystis pyrifera* extracts and/or bacterial inoculation. This pattern persisted in the final evaluation, with biostimulant-treated plants maintaining less negative Ψ_stem_ values than the control.

In the DRY trial (**Figure 3B**), all treatments displayed a similar water status at the time of the first biostimulant application, with Ψ_stem_ values ranging from –0.55 to –0.65 MPa. Following the onset of deficit irrigation on March 18, Ψstem decreased across treatments. At this date, plants inoculated with *Pseudomonas koreensis* AG-97 maintained higher Ψ_stem_ values (approximately –0.55 MPa) compared to the control, which reached values close to –0.70 MPa. At the final evaluation, most treatments exhibited further decreases in Ψ_stem_ as substrate moisture approached 0.25 m³ m^-^³. In contrast, treatment A1 maintained less negative values (approximately –0.60 MPa), differing from both A2 and the control. The control treatment reached the most negative Ψ_stem_ values, close to –1.0 MPa and was significantly different from the remaining treatments.

#### 3.2.2 Stomatal conductance

**Figure 4:**
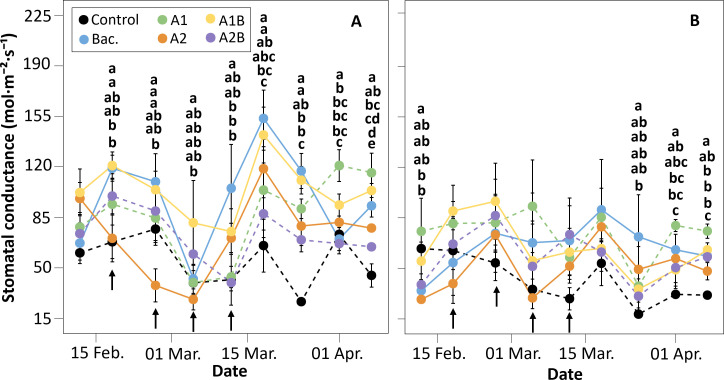
Stomatal conductance (mol·m^-^²·s^-^¹) measured in cherry rootstock ‘Colt’ under two irrigation regimes: **(A)** WET (maintained at container capacity) and **(B)** DRY (irrigated at 50% container capacity from week four). Treatments included Control (no application), Bac. (*Pseudomonas koreensis* AG-97), A1 and A2 (A1: mixed extraction enzymatic hydrolysis + alkaline; A2: alkaline extraction), and combined treatments A1B and A2B (extracts plus bacterial inoculation). Different letters indicate statistically significant differences between treatments according to Fisher’s LSD test (p < 0.05).

Under WET conditions, significant differences in stomatal conductance (g_s_) were detected following the application of biostimulants (**Figure 4A**). During the last four evaluations (March 15 to April 1), treatments receiving *Macrocystis pyrifera* extracts and/or bacterial inoculation showed significantly higher g_s_ values than the control. The combined treatment A1B consistently exhibited the highest g_s_ values during this period. Plants inoculated with *Pseudomonas koreensis* alone also showed increased g_s_, reaching values close to 155 mmol m^-^² s^-^¹. Treatment A1 showed a tendency toward higher g_s_ values during the final evaluations. In the DRY trial (**Figure 4B**), g_s_ declined in all treatments following the onset of water restriction. The control treatment consistently exhibited lower g_s_ values compared to the remaining treatments, although statistically significant differences were detected only during the last two evaluations. During this period, treatment A1 showed the highest g_s_ values, exceeding 70 mmol m^-^² s^-^¹.

**Figure 5:**
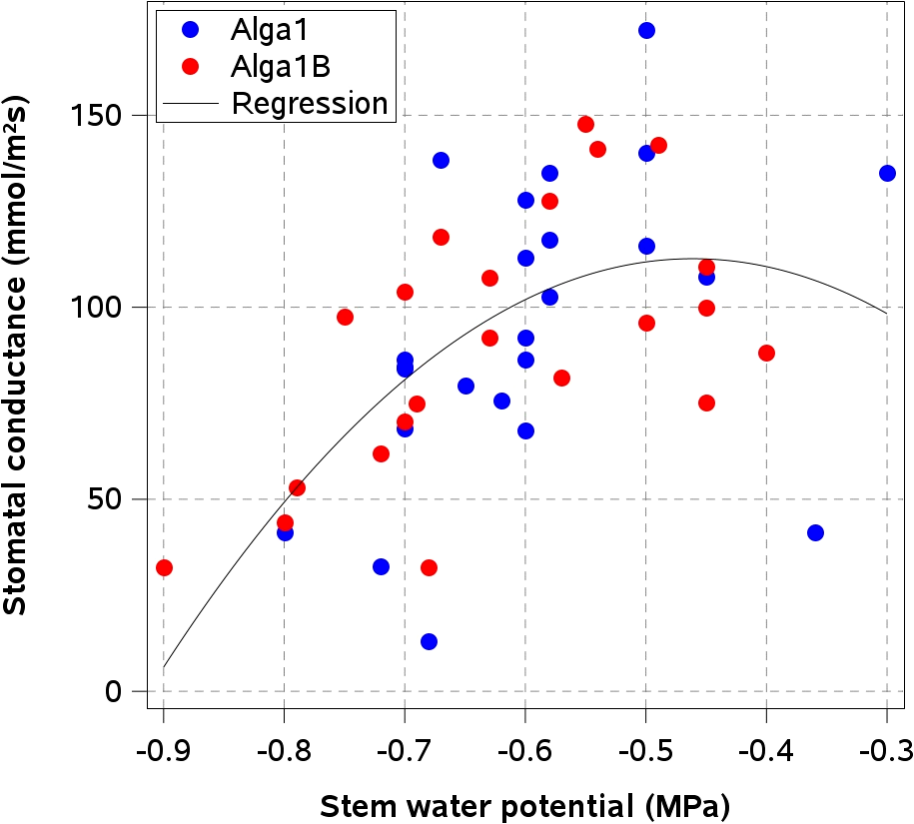
Relationship between stem water potential and stomatal conductance in cherry rootstock Colt under treatments with *Macrocystis pyrifera* extract obtained by mixed hydrolysis + Alkaline (A1) and its combination with *Pseudomonas koreensis* strain AG-97 (A1B). A second-degree polynomial regression was fitted to the data (R² = 0.36, P < 0.001), with the equation y = –551.9 x² – 508.9 x – 4.66.

A significant relationship between Ψ_stem_ and stomatal conductance was observed in plants treated with the mixed-hydrolysis *Macrocystis pyrifera* extract (A1) and its combination with *Pseudomonas koreensis* strain AG-97 (A1B) (**Figure 5**). For both treatments, g_s_ increased as Ψ_stem_ became less negative. A second-degree polynomial regression fitted to the combined dataset of A1 and A1B treatments was statistically significant (R² = 0.36, P < 0.001), describing the relationship between Ψ_stem_ and g_s_ across the evaluated range of water status.

#### 3.2.3 Photosystem II efficiency

**Figure 6:**
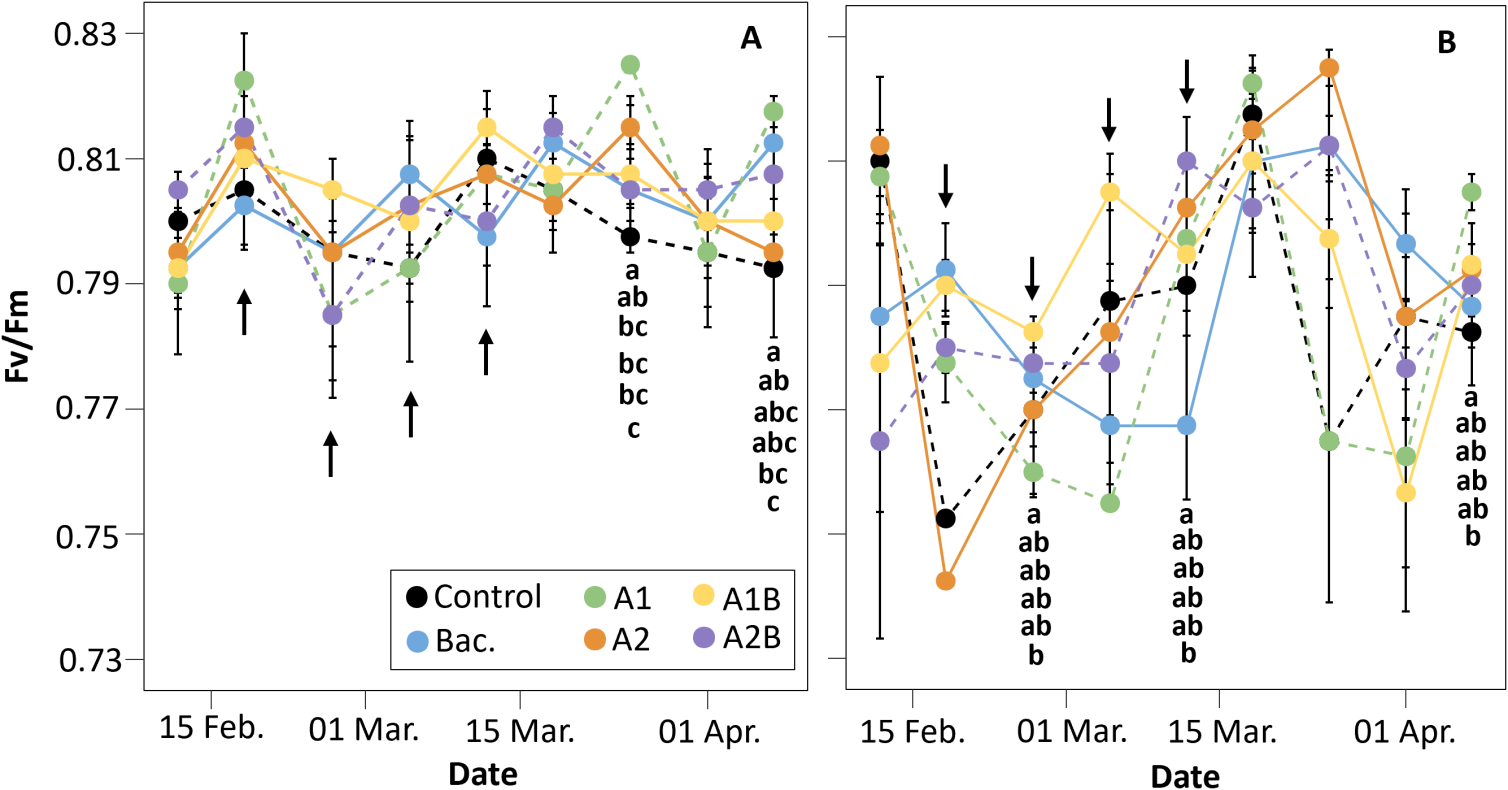
Photosynthetic efficiency (*Fv*/*Fm*) measured in cherry rootstock ‘Colt’ under two irrigation regimes: **(A)** WET (maintained at container capacity) and **(B)** DRY (irrigated at 50% container capacity from week four). Treatments included Control (no application), Bac. (Pseudomonas koreensis AG-97), A1 and A2 (A1: mixed extraction enzymatic hydrolysis + alkaline; A2: alkaline extraction), and combined treatments A1B and A2B (extracts plus bacterial inoculation). Black arrows indicate biostimulant application dates. Different letters indicate statistically significant differences between treatments according to Fisher’s LSD test (p < 0.05).

Photosynthetic efficiency (*Fv*/*Fm*) exhibited contrasting temporal patterns between irrigation regimes. Under WET conditions (**Figure 6A**), *Fv*/*Fm* values remained relatively stable throughout the experiment, fluctuating between 0.79 and 0.82. Following the application of *Macrocystis pyrifera* extracts, treatments A1 and A2 showed significantly higher *Fv*/*Fm* values than the control at five weeks, coinciding with a period of reduced PPFD. At the final evaluation, treatments A1 and Bac. exhibited higher *Fv*/*Fm* values compared to the control (p < 0.05). Under DRY conditions (**Figure 6B**), *Fv*/*Fm* displayed greater temporal variability. Treatments A1 and A1B showed higher *Fv*/*Fm* values than the control during mid-March, with statistically significant differences observed at several evaluation dates. Overall, treatments receiving combined applications (A1, A1B and A2B) tended to maintain *Fv*/*Fm* values comparable to or higher than the other treatments, although the timing of these differences varied. At the final evaluation, treatment A1 recorded significantly higher *Fv*/*Fm* values than the control.

#### 3.2.4 Leaf temperature

**Figure 7:**
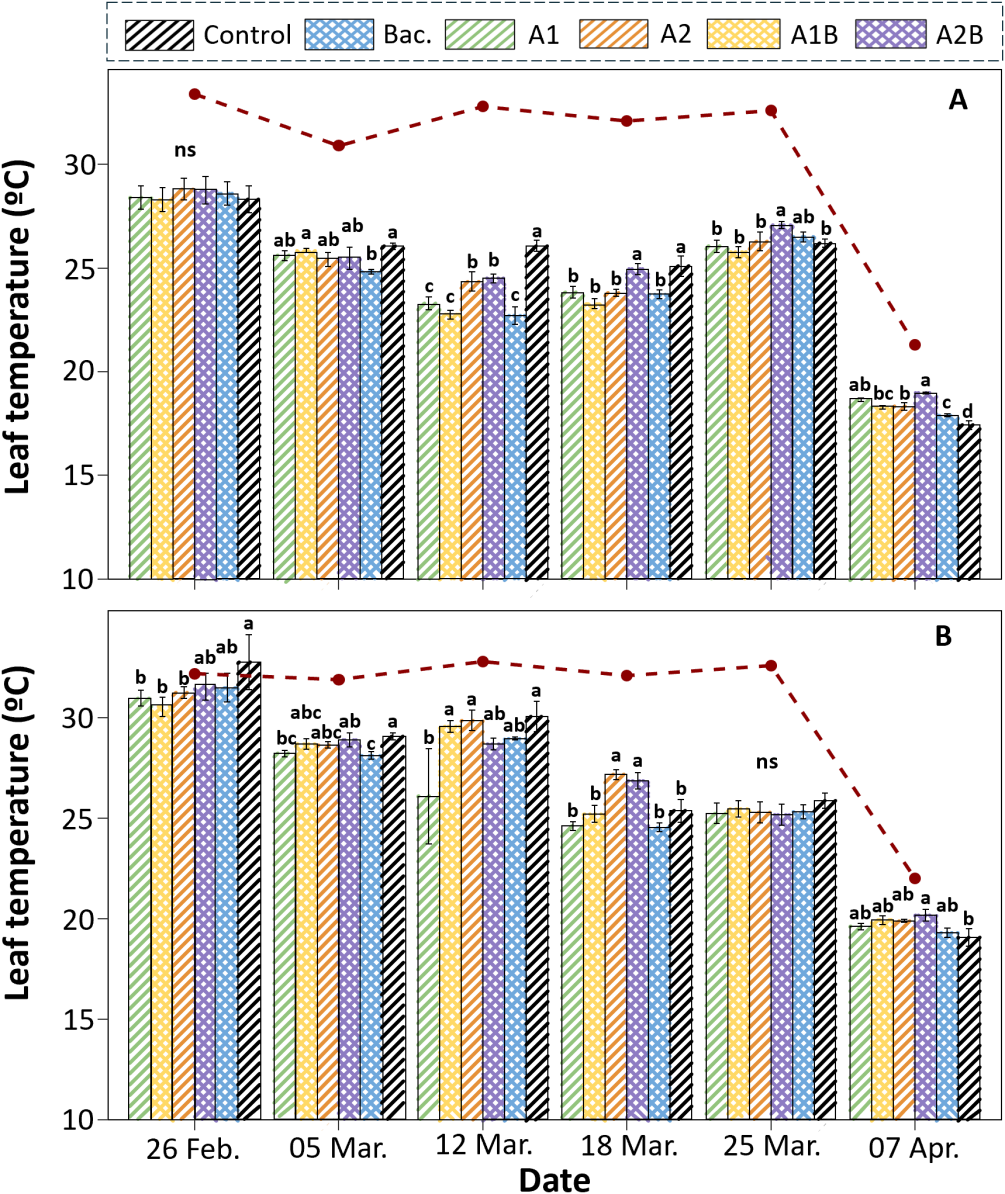
Leaf temperature (°C) of ‘Colt’ plants under two irrigation regimes: **(A)** WET (maintained at container capacity) and **(B)** DRY (irrigated at 50% container capacity from week four), measured from February 26 to April 7. Bars represent six treatments: Control (no application), Bac. (*Pseudomonas koreensis* AG-97), A1 and A2 (A1: mixed extraction enzymatic hydrolysis + alkaline; A2: alkaline extraction), and combined treatments A1B and A2B (extracts plus bacterial inoculation). The red dashed line indicates the average air temperature recorded on each date. Error bars represent standard error, and lowercase letters denote statistical differences among treatments (p < 0.05); ns indicates non-significant differences.

Leaf temperature differed among treatments and irrigation regimes over the evaluation period. Under WET conditions (**Figure 7A**), leaf temperature generally followed the temporal pattern of air temperature, with higher values recorded during periods of elevated atmospheric demand. At several evaluation dates, significant differences among treatments were observed. During periods of high air temperature, the control treatment frequently exhibited higher leaf temperatures than plants receiving *Macrocystis pyrifera* extracts and/or bacterial inoculation. At specific dates, treatments A1 and A1B showed leaf temperatures comparable to the bacterial inoculation treatment, whereas A2 and A2B displayed intermediate values. Toward the end of the evaluation period, when the air temperature decreased, differences among treatments were reduced or not significant.

Under DRY conditions (**Figure 7B**), leaf temperature also varied among treatments and dates. During the early phase of the restriction period, leaf temperature values were generally close to or above air temperature, with the control treatment showing the highest values at several dates. Significant differences among treatments were detected at specific evaluations. Treatment A1 tended to show lower leaf temperatures than the control. As atmospheric demand decreased toward the end of the experiment, leaf temperature declined across all treatments, although some differences among treatments were still observable at certain evaluation dates.

### 3.3 Morphology and biomass

#### 3.3.1 Steam growth

**Figure 8:**
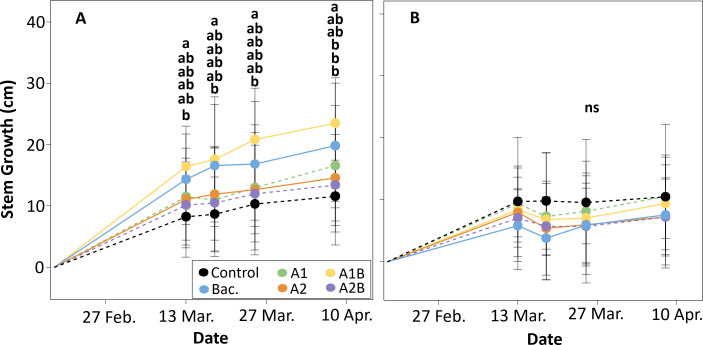
Stem growth (cm) in cherry rootstock ‘Colt’ under two irrigation regimes: **(A)** WET (maintained at container capacity, 0.33 m³·m^-^³) and **(B)** DRY (irrigated at 50% container capacity from week four), expressed relative to the first measurement. Treatments included Control (no application), Bac. (*Pseudomonas koreensis* AG-97), A1 and A2 (A1: mixed extraction enzymatic hydrolysis + alkaline; A2: alkaline extraction), and combined treatments A1B and A2B (extracts plus bacterial inoculation). Black arrows indicate biostimulant application dates. Lowercase letters above bars denote statistical differences among treatments (p < 0.05).

In the WET trial, stem growth increased steadily throughout the evaluation period relative to the first measurement (**Figure 8A**). Significant differences among treatments became evident from mid-experiment onward. Approximately four weeks after the first application of *Macrocystis pyrifera* extracts, plants receiving the combined treatment A1B exhibited the greatest stem elongation, reaching increases of nearly 17 cm, whereas the control showed an increase of approximately 8 cm. This pattern was maintained in subsequent evaluations. At the end of the trial, A1B reached an average stem growth of about 23 cm, which was significantly higher than the control (approximately 10 cm). The bacterial inoculation treatment (Bac.) showed intermediate values, with stem growth close to 19 cm.

In the DRY trial, stem growth was substantially reduced compared with the WET trial (**Figure 8B**). No significant differences among treatments were detected throughout the evaluation period. All treatments showed limited stem elongation following the onset of water restriction, indicating a generalized reduction in growth under deficit irrigation conditions.

#### 3.3.2 Biomass and biomass allocation

Under WET conditions, significant differences among treatments were observed in biomass allocation (**Table 3**). In terms of absolute dry weight, treatment A1 exhibited the lowest leaf biomass compared with the control and A2, with differences of approximately 0.05–0.06 g. In contrast, A1 showed the highest root dry weight, exceeding A2 by approximately 0.08 g. No significant differences among treatments were detected for stem dry weight, which ranged between 0.23 and 0.27 g. Relative biomass allocation also differed among treatments under WET conditions (**Table 3**). The control and A2 treatments showed the highest proportion of leaf biomass, reaching up to 25% in A2, whereas A1 exhibited the lowest percentage in this component. Root biomass accounted for more than 50% of total dry weight in most treatments, with A1 showing the highest allocation (55.7%) and A2 the lowest (48.4%). Stem biomass represented between 23% and 26% of total dry weight across treatments, with no clear differences among treatments. Under DRY conditions, no significant differences among treatments were detected for either absolute dry weight or relative biomass allocation in leaves, stems, or roots (**Table 3**).

**Table 3:** Absolute and relative dry weights of leaves, stems, and roots in cherry rootstock ‘Colt’ under two irrigation regimes: WET (maintained at container capacity, 0.33 m³ m^-^³) and DRY (irrigated at 50% container capacity from week four).

	Absolute dry weight (g)			Relative dry weight (%)		
Treatment	Leaves	Stem	Roots	Leaves	Stem	Roots
WET						
Control	0.24 ± 0.02 a	0.26 ± 0.04	0.50 ± 0.06 ab	23.9% ± 2% a	26.0% ± 4%	50.1% ± 6% ab
Bac.	0.23 ± 0.02 ab	0.23 ± 0.01	0.54 ± 0.02 ab	23.0% ± 2% ab	23.3% ± 1%	53.8% ± 2% ab
A1	0.19 ± 0.02 b	0.24 ± 0.01	0.56 ± 0.03 a	19.9% ± 2% b	24.5% ± 1%	55.7% ± 3% a
A2	0.25 ± 0.02 a	0.27 ± 0.02	0.48 ± 0.03 b	25.0% ± 2% a	26.6% ± 2%	48.4% ± 3% b
A1B	0.21 ± 0.04 ab	0.24 ± 0.03	0.54 ± 0.06 ab	21.6% ± 4% ab	24.0% ± 3%	54.4% ± 6% ab
A2B	0.23 ± 0.04 ab	0.24 ± 0.05	0.53 ± 0.08 ab	23.3% ± 4% ab	23.9% ± 5%	52.8% ± 8% ab
DRY						
Control	0.21 ± 0.12	0.36 ± 0.09	0.43 ± 0.08	20.7% ± 12%	36.9% ± 9%	43.4% ± 8%
Bac.	0.16 ± 0.12	0.38 ± 0.18	0.46 ± 0.09	16.3% ± 12%	37.8% ± 18%	46.0% ± 9%
A1	0.22 ± 0.08	0.39 ± 0.15	0.39 ± 0.11	22.1% ± 8%	38.5% ± 15%	39.4% ± 11%
A2	0.22 ± 0.06	0.28 ± 0.08	0.5 ± 0.08	21.8% ± 6%	28.1% ± 8%	50.1% ± 8%
A1B	0.15 ± 0.18	0.43 ± 0.17	0.43 ± 0.07	14.9% ± 18%	42.5% ± 17%	42.6% ± 7%
A2B	0.15 ± 0.12	0.47 ± 0.20	0.38 ± 0.08	14.8% ± 12%	47.3% ± 20%	38.0% ± 8%

Treatments included Control (no application), Bac. (*Pseudomonas koreensis* AG-97), A1 and A2 (A1, mixed extraction enzymatic hydrolysis + alkaline; A2, alkaline extraction), and combined treatments A1B and A2B (extracts plus bacterial inoculation). Data are presented as mean ± standard deviation (SD). Different lowercase letters indicate significant differences among treatments according to Fisher’s LSD test (p < 0.05).

### 3.4 Carbon assimilation and isotopic discrimination

**Figure 9:**
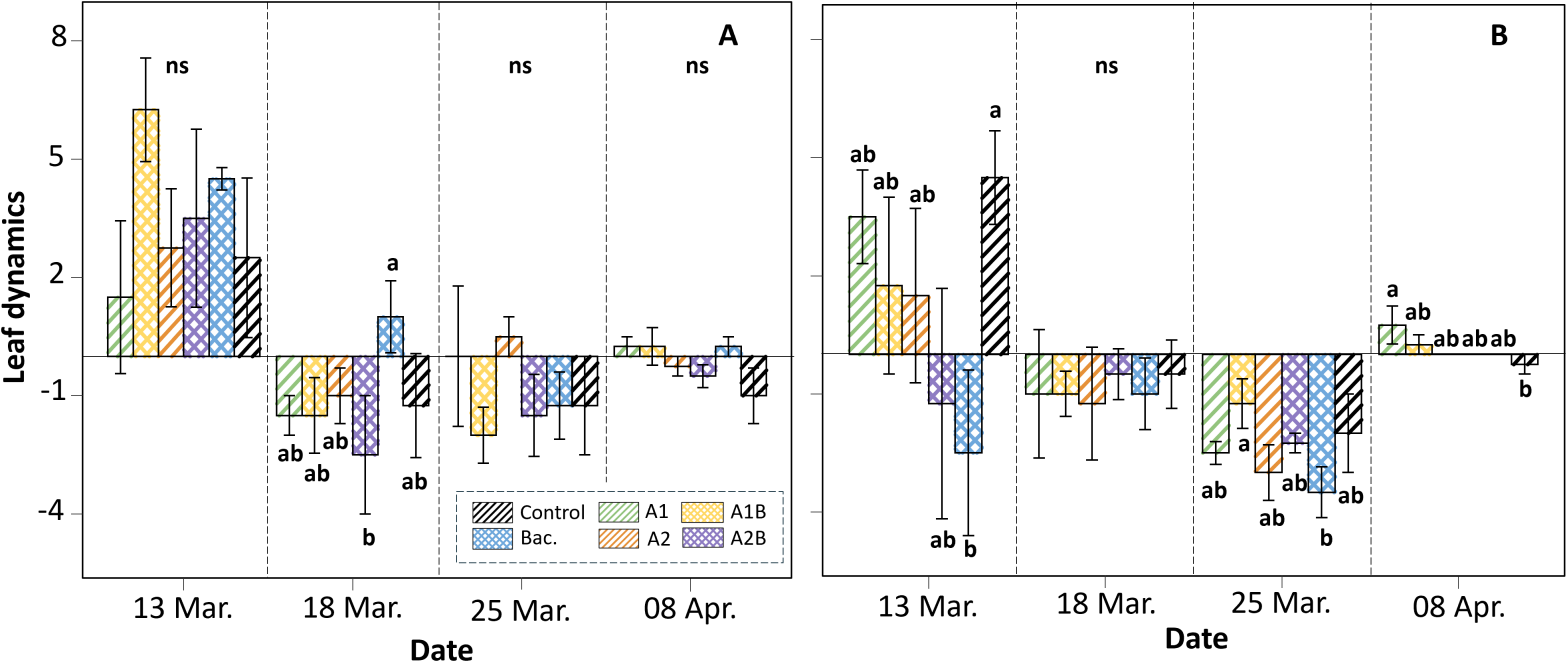
Leaf dynamics (change in number of leaves relative to the previous measurement) in ‘Colt’ plants under two irrigation regimes: **(A)** WET (maintained at container capacity, 0.33 m³·m^-^³) and **(B)** DRY (irrigated at 50% container capacity from week four), evaluated between March 13 and April 8. Bars represent six treatments: Control (no application), Bac. (*Pseudomonas koreensis* AG-97), A1 and A2 (A1: mixed extraction enzymatic hydrolysis + alkaline; A2: alkaline extraction), and combined treatments A1B and A2B (extracts plus bacterial inoculation). Error bars indicate standard error, and lowercase letters denote statistical differences among treatments according to Fisher’s LSD test (p < 0.05); ns indicates non-significant differences.

**Figure 10:**
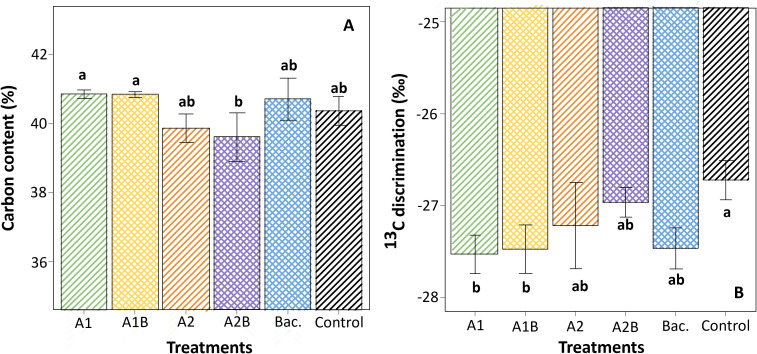
**(A)** Carbon content (%) and **(B)** ¹³C discrimination in cherry rootstock ‘Colt’ under different treatments in the WET trial. Treatments included A1 and A2 (A1, mixed extraction enzymatic hydrolysis, + alkaline; A2, alkaline extraction), Bac. (*Pseudomonas koreensis* AG-97), combined treatments A1B and A2B (extracts plus bacterial inoculation), and Control (no application). Error bars represent standard error, and different lowercase letters indicate significant differences among treatments according to Fisher’s LSD test (p < 0.05).

**Figure 11:**
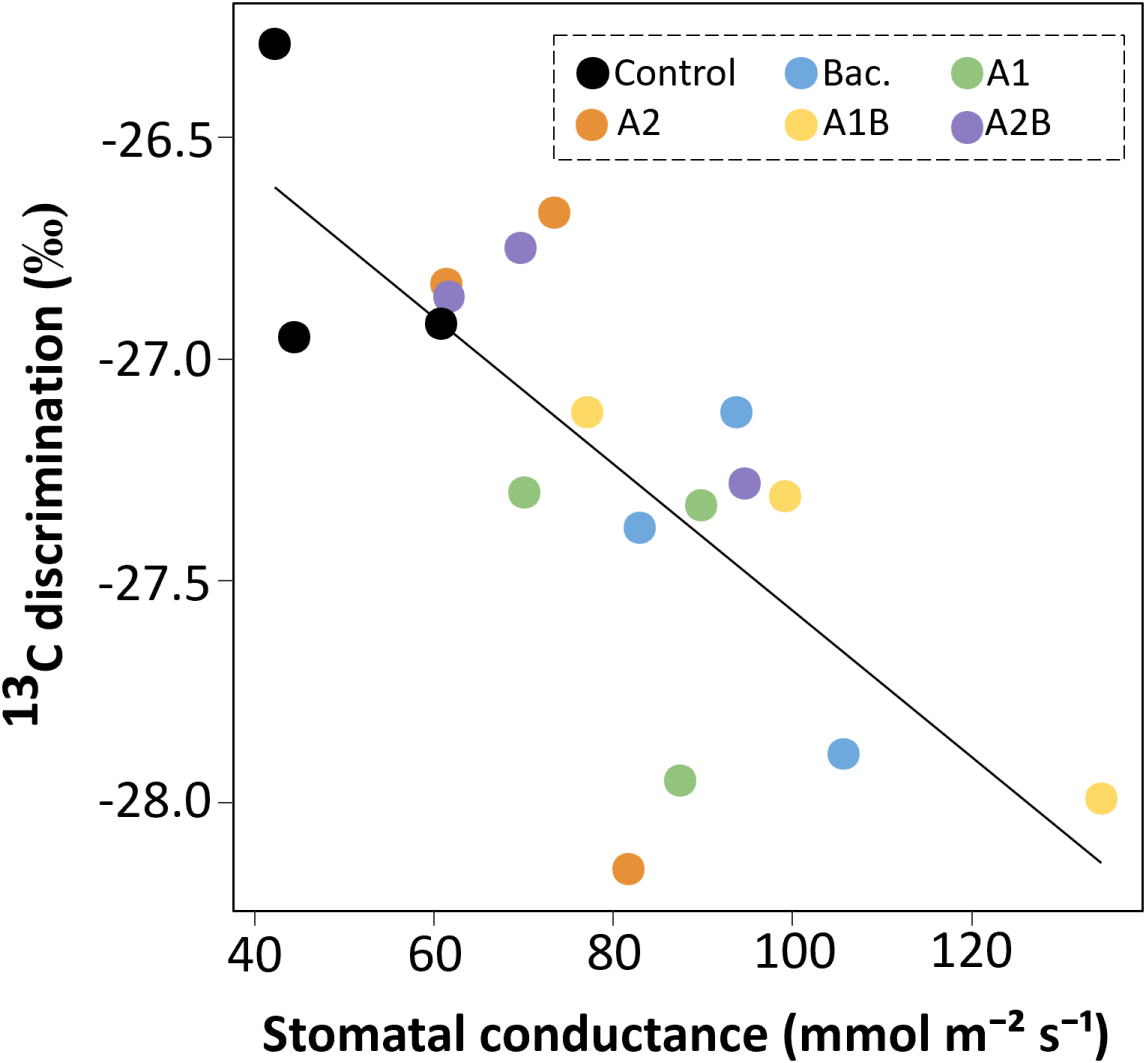
Relationship between stomatal conductance (mmol m^-^² s^-^¹) and ¹³C discrimination in cherry rootstock ‘Colt’ under different treatments. The fitted model is described by the equation y = -25.91 − 0.02x (R² = 0.54; p < 0.01). Treatments included Control (no application), Bac. (*Pseudomonas koreensis* AG-97), A1 and A2 (A1, mixed extraction enzymatic hydrolysis, + alkaline; A2, alkaline extraction), and combined treatments A1B and A2B (extracts plus bacterial inoculation).

Under WET conditions, significant differences in leaf carbon content were observed among treatments (**Figure 9A**). Treatments A1 and A1B exhibited the highest carbon percentages, exceeding 42%, whereas treatment A2B showed the lowest values, below 40%. Carbon isotope discrimination (δ¹³C) also differed among treatments under WET conditions (**Figure 9B**). The most negative δ¹³C values (approximately −27.5‰) were recorded in treatments A1 and A1B, while the control exhibited the least negative values (approximately −26‰). Under DRY conditions, no significant differences among treatments were detected for either leaf carbon content or δ¹³C (data not shown). A significant relationship between stomatal conductance and carbon isotope discrimination was observed across treatments (**Figure 10**). A linear regression analysis (**Figure 11**) showed that δ¹³C values became more negative as stomatal conductance increased, according to the fitted model y = −25.91 − 0.02x (R² = 0.54; p < 0.01). Treatments exhibiting higher stomatal conductance tended to cluster toward more negative δ¹³C values, whereas the control treatment was associated with lower conductance and less negative δ¹³C values.

### 3.5 Compatibility of macrocystis extracts and Pseudomonas spp. populations

High compatibility was observed between *Macrocystis pyrifera* extracts and the strain *Pseudomonas koreensis* AG-97 (**Table 4**). Both extracts, A1 (combined enzymatic and alkaline hydrolysis) and A2 (alkaline extraction), supported bacterial growth under *in vitro* conditions.

**Table 4:** Microbial count of *Pseudomonas* spp. obtained from the *in vitro* evaluation with *Macrocystis pyrifera extracts*.

Treatment	Bacterial count (Log_10_ CFU mL^-1^)
*Pseudomonas koreensis* AG-97	8.50
*Pseudomonas koreensis* AG-97 and A1	8.11
*Pseudomonas koreensis* AG-97 and A2	8.32

*A1 corresponds to extract of *Macrocystis pyrifera* obtained via mixed hydrolysis + alkaline; A2 corresponds to the extract of *Macrocystis pyrifera* obtained via alkaline.

Result expressed in Colony Forming Units (Log10 CFU mL^-1^).

In planta quantification of cultivable *Pseudomonas* spp. populations in the rhizosphere revealed no significant differences among treatments under either irrigation regime (WET or DRY) (**Table 4**).

## 4 Discussion

The seaweed biostimulant A1, derived from a combination of enzymatic and alkaline hydrolysis of *Macrocystis pyrifera* (i.e. mixed hydrolysis), showed the greatest consistency in improving the drought tolerance of young ‘Colt’ rootstock plants, regardless of inoculation with the AG-97 strain of *Pseudomonas koreensi*s. Improving the drought tolerance of the ‘Colt’ rootstock is paramount for the sustainability of the Chilean sweet cherry industry, as this genotype is highly sensitive to water deficits (Bouljan and Claverie, 2004) and accounts for almost 40% of commercial sweet cherry orchards (Masman, 2024). The results of the present study confirmed the low tolerance of the ‘Colt’ rootstock to drought conditions, as Control plants exhibited no reduction in stomatal conductance in response to a substantial decrease in soil water content and plant water status, as it was evidenced by the absence of a significant correlation between stem water potential (Ψ_stem_) and stomatal conductance (g_s_) throughout the experimental period. Under dry conditions, withholding irrigation reduced substrate moisture from the container capacity (θCC ~0.35 m³ m^-^³) to values near the permanent wilting point (θ_PWP_ ~0.15 m³ m^-^³) over three consecutive one-week periods. A lack of effective stomatal control to reduce transpiration when atmospheric evaporative demand remained high (2.0-3.5 kPa) caused the largest drop in Ψ_stem_ in Control plants among the treatments.

While Control plants experienced a maximum water stress severity of -1.1 MPa, A1 plants exhibited a Ψ_stem_ value of -0.7 MPa, which was very close to the optimum plant water status for *Prunus* spp. for the environmental conditions of early April, according to the Ψ_stem_ baseline developed by McCutchan and Shackel (1992). Furthermore, in the WET trial, where the substrate moisture was continuously maintained at very high values (between container capacity and saturation, θ_SAT_ ~0.47 m³ m^-^³), Ψ_stem_ was consistently above -0.7 MPa, confirming that the application of mixed-extracted seaweed biostimulants (A1 and A1B) successfully maintained ‘Colt’ plants under optimum water conditions. Notably, most treatments exhibited higher plant water status than the Control at the final measurement date, except for the alkaline-extracted seaweed biostimulant (A2). In this study, A2 plants registered the lowest Ψ_stem_ and root dry weight among all treatments. While differences in root dry weight were only detectable under WET conditions, differences in Ψ_stem_ were found under DRY conditions. In the present study, plants subjected to the DRY regime were similarly irrigated to those in the WET trial for 82% of the evaluation period, including the period of highest seasonal root growth rates (spring, October to November). This indicates that A2 plants exhibited reduced root development under well-irrigated conditions, which could have affected water uptake in the DRY plot, where water availability was limited. The absence of substantial treatment effects on root dry weight under DRY conditions does not indicate that biostimulants had no effect on root development and growth. These results suggest that DRY conditions were severe enough to reduce root carbon allocation in all treatments. In fact, root dry weight across all treatments under DRY conditions was 20% less than under WET conditions.

In this context, one of the most important traits for drought tolerance is the ability to close stomata when soil water content is low. The application of both mixed-extracted seaweed biostimulants, A1 and A1B, induced stomatal closure when the severity of plant water stress increased. This was evident by analyzing the significant quadratic relationship between Ψ_stem_ and gs (P<0.0001; R^2^ = 0.36). As this study was conducted under semi-shading conditions, the relationship between Ψ_stem_ and g_s_ was not as strong as has been reported for sunlit leaves in other species of *Prunus* spp (Spinelli, 2016). When A1 and A1B-treated plants showed Ψ_stem_ values close to –1.0 MPa, g_s_ was reduced to almost zero, limiting transpiration and plant dehydration under the DRY conditions. These results may indicate that mixed−extracted seaweed biostimulants were associated with stronger stomatal regulation as water stress intensified, consistent with previously reported drought−mitigation effects (Grammenou et al., 2023). The chemical analyses of seaweed extracts showed that mixed-extracted biostimulants had 73% and 42% higher concentrations of betaines and mannitol, respectively, than the alkaline-extracted biostimulants (A2 and A2B). In *Fraxinus excelsior* L., leaves with higher concentrations of mannitol were found to maintain measurable stomatal conductance under very severe water stress (pre-dawn LWP of -6.0 MPa) (Guicherd et al., 1997). In olive trees (*Olea europea* L.), two cultivars with contrasting mannitol concentrations in their leaves exhibited different stomatal behavior in response to high evaporative demand and one month of no irrigation (Lo Bianco and Avellone, 2014). However, in transgenic wheat, the amount of mannitol accumulated in leaves was found to be insufficient to protect against stress through osmotic adjustment (Abebe et al., 2003). This suggests that mannitol-induced exposure to mild osmotic perturbation may have acted as a priming stimulus, promoting anticipatory activation of protective mechanisms such as proline accumulation and improved stomatal control. Therefore, it is plausible that mannitol-richer algal extracts, A1 and A1B, may have induced a physiological priming effect that enhances stomatal responsiveness and overall plant resilience under subsequent drought stress. In addition, seaweed extracts promote osmolyte accumulation and osmoprotection; their application increases proline and related osmolytes in several species, contributing to cellular osmotic adjustment during water or salt stress (Chen et al., 2021; Islam et al., 2020).

*Pseudomonas koreensis* has been shown to enhance drought tolerance and modulate ABA−related gene expression in Arabidopsis, suggesting a potential role for this PGPB in influencing stomatal ABA pathways. However, the covariance analysis showed that both mixed-extracted seaweed biostimulants exhibited the same relationship between Ψ_stem_ and g_s_. This finding may indicate that the AG-97 strain of *Pseudomonas koreensis* was not responsible for inducing the stomatal closure at low Ψ_stem_ in A1-treated plants, but this does not exclude microbial influence on stomatal signaling. In fact, in the present study, the inoculation with AG-97 strain, either alone or combined with a seaweed biostimulant, enhanced g_s_ under well and deficit-irrigated conditions. Under WET conditions, the BAC and A1B treatments exhibited 2- to 3-fold higher gs than the Control for 70% of the evaluation period. Under DRY conditions, the BAC biostimulant was the only treatment that consistently showed higher g_s_ than the Control. Regardless of the irrigation strategy, the greater effect of the AG-97 strain on g_s_ was detected one month after inoculation. In the last two measurements (first week of April), a decrease in g_s_ was observed in the BAC plants, although these treatments continued to show significantly higher g_s_ values than the Control. The strain AG-97 has been identified as a producer of indole acetic acid (IAA) (Reyes-Castillo et al., 2019), and can enhance root growth in herbaceous crops (Reyes-Castillo et al., 2019; Sepúlveda-Caamaño et al., 2018) and to stimulate water stress tolerance in a *Prunus* sp. rootstock (López-Fernández, 2024). High concentrations of IAA have been associated with increased stomatal conductance in plants by stimulating ethylene synthesis, which inhibits ABA-mediated stomatal closure (Tanaka et al., 2006). This provides a plausible explanation for the higher g_s_ observed under high moisture and the reduced stomatal responsiveness of AG−97 inoculated plants under moderate water deficit. Additionally, the strain’s lower stimulatory effect on stomatal opening at the end of the WET and DRY tests may be due to decreased bacterial activity as autumn approaches, coinciding with reduced ambient and, therefore, soil temperatures. In general, mesophilic bacteria such as *Pseudomonas koreensis* exhibit maximal metabolic activity and growth at temperatures of 30 °C (BacDive, 2026). In April, the maximum temperature fell to 20 °C; therefore, the soil in the trial is estimated to have been near 16 °C, which is below the optimal temperature for this type of PGPB. Consequently, these results indicate that although AG−97 did not alter the regression pattern between Ψ_stem_ and g_s_, inoculation stimulated g_s_, likely through IAA−ethylene signaling and metabolic enhancement modulated by seasonal soil temperature.

Inoculation of the AG-97 strain of *Pseudomonas koreensis* in WET plants treated with mixed seaweed extracts (A1B) produced a 200% increase in stem elongation compared to the control treatment. Greater stem elongation is relevant in environments with moderate to low light intensity, as in this study, as it favors light interception. These results demonstrate that strain AG-97 increased plant height in A1-treated plants, as no significant difference in stem length was observed between A1 and Control. Although A1B had a higher stem length, the dry weight of the stem was similar in all treatments, indicating that the increase in elongation was not due to greater carbohydrate accumulation. Under conditions of high-water availability (Ψ_stem_ > –0.6 MPa), organ growth depends mainly on turgor, which allows elongation without necessarily increasing dry biomass (Taiz et al., 2017; Blum, 2017). It should be noted that no variation in Ψ_stem_ was observed between treatments under humid conditions, suggesting that the increase in stem length was probably not associated with greater water accumulation. Therefore, the greater elongation in A1B plants may be due to more elastic cell walls, since softer walls with equal turgidity may exhibit higher growth rates. Several studies have addressed the impact of seaweed extracts and PGPB on cell wall loosening, suggesting that organic compounds such as polyphenols, polysaccharides, and hormones (e.g., IAA) could stimulate the synthesis of cell wall-softening proteins such as expansins, which would explain the greater stem length in A1B plants (Majda and Robert, 2018).

Despite A1 plants showed no higher stem length than Control, the WET treatment seems to have changed the allocation of carbohydrates compared to A2. Thus, A1 plants accumulated less dry mass in leaves than A2, but more in roots at the end of the experiment. Surprisingly, A1 and A1B exhibited 2% higher percentage of total carbon in leaves than A2. The percentage of carbon (%C) measured by IRMS quantifies the proportion of carbon atoms in a homogenized, dried sample and is not influenced by tissue thickness, leaf mass area, or mineral elements, but only by the relative abundance of carbon-containing molecules. Oven-drying at 60 °C for 72 hours removes water but retains all mineral ashes and structural compounds. Consequently, the resulting dry mass reflects not only carbon-rich organic matter but also non-carbon mineral content and structural density. Therefore, A1 leaves were likely to be thinner, less dense, and to contain a lower mineral ash content, which results in a lower oven-dry mass than A2 leaves. This means that A1 leaves probably exhibited a higher proportion of carbon-rich compounds, such as soluble carbohydrates, phenolics, cuticular lipids, or lignin, than A2 leaves. The lower leaf construction costs of A1 plants indicate reductions in carbon and mineral investment in foliage. This suggests a reallocation of resources toward belowground growth, consistent with the observed 7% higher relative root dry biomass in A1 compared with A2. Although not directly measured, such a shift in resource allocation could contribute to enhanced root development and, potentially, greater water uptake capacity.

Although plants treated with A1 and A1B showed greater drought tolerance due to more efficient stomatal closure, surprisingly, these treatments also had the lowest intrinsic water use efficiency, as assessed by ^13^C isotope discrimination in leaves under WET conditions, but not under DRY conditions. The fact that the Control in the WET trial exhibited greater water use efficiency is closely related to its lower stomatal conductance throughout the experiment. Under conditions of high substrate moisture between field capacity and saturation, it is possible that the plants experienced periods of root hypoxia, so maintaining high stomatal conductance is key to drying the soil and improving aeration. In addition, during the warmest months of the trial (February-March), the leaf temperature of the Control plants was 1–1.5 °C higher than that of the treatments with biostimulants, particularly A1B. These results clearly indicate that maintaining high transpiration rates in plants treated with biostimulants, in a very moist substrate and at temperatures near 30 °C, is a positive attribute, even if water use efficiency is low. Surprisingly, the lower stomatal conductance of the Control plants was not low enough to reduce carbon assimilation in the leaves. This was reflected in a higher dry matter percentage in the Control leaves, approximately 20% higher than in the A1 plants in the WET trial. Given that the Control plants ended the trial with the fewest leaves, this greater accumulation of dry matter would correspond to the distribution of the same amount of carbohydrates in fewer sinks (leaves). The study was conducted in a shade house covered with 20% shade cloth, which means that the plants grew under an average light intensity 50% lower than the saturation point for *Prunus* spp. (600 µmol m^-^² s^-^¹) (De Jong, 1983). For this reason, the fact that the leaves of the Control were denser than those of the A1 plants could have generated greater resistance to CO_2_ diffusion through the mesophyll, affecting photosynthetic efficiency. In contrast, the less dense leaves of the A1 plants favor their expansion to capture light and dissipate heat. This would explain why the A1 plants exhibited slightly higher *Fv*/*Fm* values than the Control plants at the end of the experiment, both in the WET and DRY tests.

## 5 Conclusions

The present study shows that the mixed-hydrolysis seaweed biostimulant A1 was the most effective treatment for enhancing drought tolerance in young ‘Colt’ rootstocks. This genotype is highly sensitive to water deficits and key to the sustainability of the Chilean sweet cherry industry. The A1 demonstrated consistent effectiveness in enhancing plant water status under drying conditions. This enabled ‘Colt’ plants to maintain optimal stem water potential values for *Prunus* spp., while effectively promoting stomatal closure as soil moisture levels decreased. This improved stomatal regulation was probably linked to the biochemical composition of mixed-extracted seaweed products, particularly their high mannitol content. Although inoculation with *Pseudomonas koreensis* AG 97 did not modify the response curve of stomatal conductance to plant water stress, this strain enhanced stomatal conductance and stimulated stem elongation under high-moisture conditions. However, these PGPB physiological effects were diluted as soil temperatures declined in autumn. Finally, A1-treated plants exhibited functional leaf traits that were advantageous under shadehouse conditions, including lower leaf dry matter content, greater leaf expansion, reduced leaf temperature, and slightly higher photosynthetic efficiency. Because oven-drying retains mineral residues, the lighter A1 leaves indicate lower construction costs, suggesting that resources were redirected below-ground. This interpretation aligns with the 7% higher relative root biomass in A1 plants and may reflect enhanced capacity for root development and water uptake. The results indicate that mixed-extracted seaweed biostimulants, either alone or in combination with PGPR inoculation, are a promising biologically grounded strategy to strengthen the drought resilience and physiological performance of ‘Colt’ rootstocks under contrasting water regimes.

## Data Availability

The datasets presented in this article are not readily available because The dataset generated during this study is part of a privately funded research project and is subject to contractual confidentiality agreements. Therefore, the full raw data cannot be publicly shared. Aggregated or derived data supporting the findings of this study are available from the corresponding author upon reasonable request and with permission from the funding company. Requests to access the datasets should be directed to arcalderon@udec.cl.
